# Radiographic evaluation of early periprosthetic acetabular bone contrast and prosthetic head acetabular coverage after uncemented and cemented total hip prosthesis in dogs

**DOI:** 10.1186/s12917-016-0900-8

**Published:** 2016-12-03

**Authors:** Ayman A. Mostafa, Karin Lucas, Ingo Nolte, Patrick Wefstaedt

**Affiliations:** 1 Small Animal Clinic, University of Veterinary Medicine, Hannover, Foundation, Bünteweg 9, 30559 Hannover, Germany; 2 Department of Surgery, Faculty of Veterinary Medicine, Cairo University, PO Box 12211, Giza, Egypt

**Keywords:** Radiographic evaluation, Periprosthetic acetabular contrast, Acetabular coverage, Total hip arthroplasty, Dogs

## Abstract

**Background:**

Coxofemoral osteoarthritis is a chronic, disabling condition affecting people and dogs, with THA providing an excellent return to function in severely affected joints. The principal role of THA is to restore an adequate range of motion to the hip joint while transferring load from the acetabulum in order to improve the survival of the implant and enhance the limb function in the short and long terms. The objectives of the study reported here were, therefore, to radiographically evaluate periprosthetic acetabular bone GV and to assess prosthetic head acetabular coverage after 4 months of uncemented and cemented THA in dogs. Means periprosthetic acetabular GV for each and combined 3 regions of interest (zones 1, 2 and 3) were calculated immediately and 4 months after THA. Prosthetic head Norberg (PHN) angle was also measured to assess the degree of prosthetic head acetabular coverage after 4 months of surgery.

**Results:**

Zones 2 and 3 showed a significant increase in the mean bone GV after 4 months of uncemented THA. No differences in zones 1–3 after 4 months of cemented THA. Combined zones showed a significant increase in overall mean bone GV 4 months after uncemented THA; whereas, no changes were identified after 4 months of cemented THA. The PHN angles did not change after 4 months of uncemented and cemented THA and did not differ significantly between the 2 designs of hip arthroplasty.

**Conclusions:**

Regional periprosthetic acetabular bone GV varies with the design of THA. None of the designs showed periprosthetic acetabular bone lucency. No differences identified in the degree of prosthetic head acetabular coverage in both designs, indicating proper implant stability after 4 months of surgery. Further longer–term investigation on larger population is however still warranted.

## Background

Canine hip dysplasia is a potentially debilitating orthopedic disease affecting large and giant breeds, with associated coxofemoral joint laxity being the main cause of secondary osteoarthritis [1]. Coxofemoral osteoarthritis is a chronic, disabling condition affecting people and dogs, with total hip arthroplasty (THA) providing an excellent return to function in severely affected joints [2–5]. The principal role of THA is to restore an adequate range of motion to the coxofemoral joint while transferring load from the acetabulum allowing early weight bearing [6]. The main goal of THA is to improve the survival of the implant and enhance the limb function in the short and long terms [7]. However, alteration in bone remodeling around the prosthetic implants may result in changes in the adjacent bone density [8]. Thus, maintenance of adequate periprosthetic bone stock is crucial, for instance, periprosthetic bone loss increases the risk of aseptic loosening, implant migration, and periprosthetic fracture [8, 9].

A homeostatic equilibrium between bone formation and bone loss is achieved during natural biomechanical loading, whereas unnatural stresses or strains induce morphological changes and alteration in bone remodeling [6, 10, 11]. After THA, a similar process occurs when abnormal stress or strain is applied to the bone surrounding the prosthetic implants [5, 10, 11]. An associated bone weakness and possible bone fracture or implant loosening and subsequent implant failure may result [5, 6, 8, 9]. On the contrary, adaptive bone remodeling may develop secondary to load transmission from the implant to the adjacent bone [12]. This may result in cortical bone thickening and increased bone contrast around the prosthetic implant.

Several veterinary studies had focused on evaluation of the periprosthetic femoral bone remodeling after uncemented and cemented designs of THA [5, 12–15]. To our knowledge, there is no a priori interest has been focused on quantitative assessment of periprosthetic acetabular bone changes after THA in dogs. A medical and radiological image processing software^4^ has been used previously to evaluate calvarial bone density in mice [16], and recently to assess periprosthetic femoral bone GV after THA in dogs [5]. In the present study, we used the same software to quantify the mean bone GV around the prosthetic acetabular cup and femoral head after uncemented and cemented THA, respectively, using VD radiographs. We propose that GV may represent regional adaptive bone remodeling of each acetabular zone around the prosthetic implant after THA in dogs. Additionally, the degree of prosthetic head acetabular coverage was evaluated after THA via calculating the Norberg angle (PHN angle) to assess implant stability after 4 months of limb use [5].

Our objectives were to evaluate the regional periprosthetic acetabular bone GV after 4 months of uncomplicated uncemented and cemented THA, and to assess prosthetic head acetabular coverage after 4 months of surgery using a clinically applicable medical and radiological image processing software. We hypothesized that regional acetabular bone adaptation and the degree of prosthetic head acetabular coverage may vary with the design of THA.

## Methods

### Dogs

A total of 14 consecutive patients admitted over a year in the Small Animal Hospital at the University of Veterinary Medicine at Hanover were enrolled in the study reported here. Medical records and pelvic radiographs of these dogs were retrieved. No ethical approval was required, as the pre- and post-operative radiographic examinations were obtained as a routine diagnostic follow-up of each patient enrolled in our study. Subjects enrolled included client-owned, large-breed dogs with a minimum body weight of 27 kg and were at least 12 months of age that had progressive unilateral pelvic limb lameness and radiographic evidence of severe osteoarthritis and subluxation of the corresponding coxofemoral joint. These dogs had unilateral uncemented^1^ and cemented^2^ hip prostheses. Additional inclusion criteria included no history of previous limb surgery and no evidence of neurologic deficits or pathologic changes in the pelvic limb other than coxarthrosis. Patients were allocated to uncemented and cemented groups (7 dogs each) and each group was investigated immediate and 4 months after THA. Dogs that had major postoperative complications, such as implant failure, fractures, or dislocations, or had bilateral THA or revision surgery were excluded from the study.

### Radiographic measurements

Extended VD radiographs of the pelvis were obtained immediately after THA and at 4 months postoperatively for each operated dog. All radiographic projections were standardized using standard exposure factors of 70–73 kV and 10 mA and a focal spot film distance (FFD) of 110 cm. Digitized radiographs were reviewed for quality and positioning by a qualified radiologist (IN). Positioning was considered satisfactory if the 2 limbs were extended and the 2 femurs were parallel to each other and to the x-ray table. Ventrodorsal projections with radiographic evidence of pelvic tilt were excluded from the study.

**Fig. 1:**
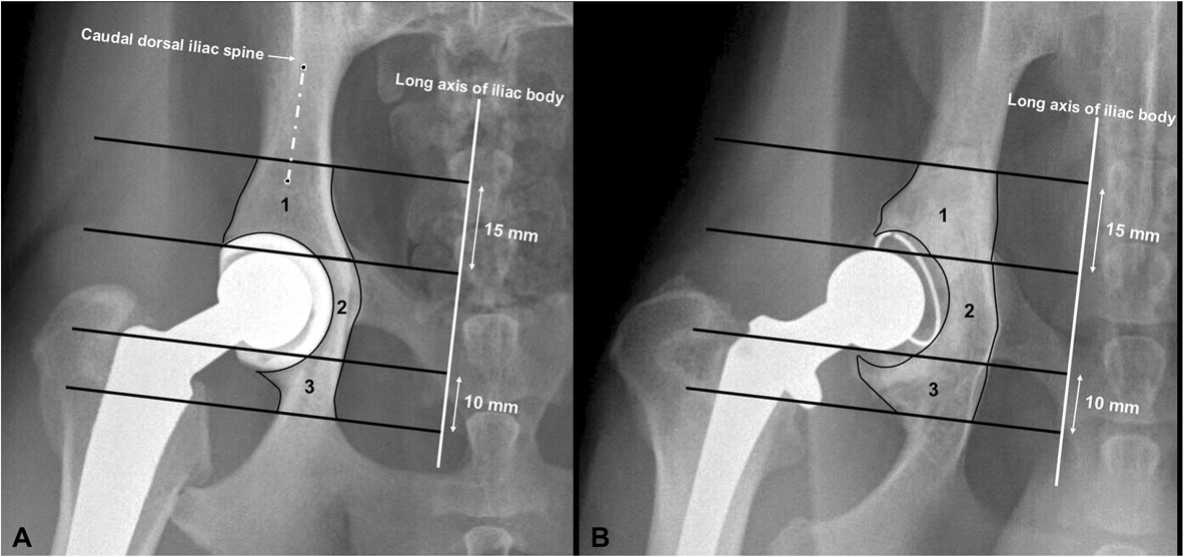
Ventrodorsal radiographic projections of canine coxofemoral joints after uncemented (**a**) and cemented (**b**) total hip arthroplasties illustrating the 3 periprosthetic acetabular zone (3-region of interest model) analysis. Zone 1 = cranial (iliac) acetabulum; zone 2 = central (pubic) acetabulum; zone 3 = caudal (ischial) acetabulum

All digitized radiographs were retrieved using PACS^3^ and a medical workstation. Digitized images were analyzed by the same investigator (AAM) using medical and radiologic image processing software^4^ with a template to create three well-defined periprosthetic acetabular zones (a 3-region of interest model), and an image magnification of × 150 (Fig. 1). Our three reported regions of interest (ROIs, zones) were modified from a four-region model previously designed for describing the areas of bone remodeling around the circumference of the acetabular component after THA in people [17–19].

The area and mean radiographic bone GV for each region were automatically calculated by the software program^4^ immediately and 4 months after THA. Assessment of bone remodeling relied on determining the mean radiographic bone contrast, expressed as mean gray scale value (GV), of each region around prosthetic acetabular cup (in uncemented THA) or prosthetic femoral head (in cemented THA) on digitized radiographs. The 3 well-defined periprosthetic acetabular zones (ROIs) were created by drawing 4 parallel transverse lines, perpendicular to the long axis of the body of the corresponding ilium. Two middle transverse lines were drawn tangential to the cranial and caudal extents of the prosthetic femoral head for each design of THA (Fig. 1). The most caudal transverse line was drawn 1 cm caudal to the caudal transverse middle line, whereas the most cranial line was drawn 1.5 cm cranial to the cranial transverse middle line (Fig. 1). The long axis of the body of the ilium was defined as the line extending at the level of the caudal extent of the caudal dorsal iliac spine and bisecting the body of the corresponding ilium (Fig. 1). Regional mean bone GV was then measured for each zone in the uncemented and cemented THA. Zone 1 represents cranial (iliac) periprosthetic acetabular region, zone 2 represents central (pubic) periprosthetic acetabular region, and zone 3 represents caudal (ischial) periprosthetic acetabular region on the VD radiographs of uncemented and cemented groups (Fig. 1).

The areas and means of all 3 acetabular zones were combined to determine the total area and overall bone GV around the prosthetic hip of each operated limb immediately and 4 months after uncemented and cemented THA. The average periprosthetic bone GV for each zone, 4 months after surgery, was also expressed as the percentage of immediate postoperative value to compare the percentage of bone GV for each zone in the uncemented and cemented groups of dogs. The selected digital image of each operated limb was analyzed by the plugins of the image processing software (*File/Open* “to select digital image”/*Magnifying glass* “for image magnification of × 150”/*Analyze/Set Scale/Set Measurements* “to select *Area* and *Mean*”/*Polygon selections* “to create a ROI (zone)” /*Plugins/Analyze / Measure and Set Label*), as previously published [5]. The analyzed images and measurements were saved for statistical analysis. The head of each prosthetic implant was used on each corresponding radiograph as a control to standardize the GV and ensure the consistency of data analysis between radiographs. This was carried out by placing a best-fit circle on each prosthetic femoral head. The area and mean GV for each circle were calculated immediately and 4 months postoperatively.

**Fig. 2:**
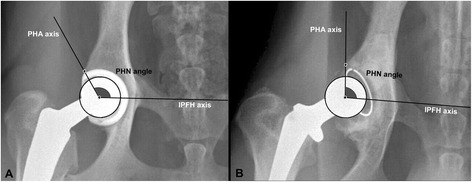
Ventrodorsal radiographic projections of canine coxofemoral joints after uncemented (**a**) and cemented (**b**) total hip arthroplasties illustrating the prosthetic head Norberg angle measurement. PHN angle = prosthetic head Norberg angle; IPFH axis = interprosthetic-femoral head axis; PHA axis = prosthetic head acetabular axis

On the same extended VD projection of each pelvis, PHN angle was measured using landmarks modified from previously established techniques [5, 20, 21]. The PHN angle was assessed to evaluate the degree of acetabular coverage immediately and after 4 months of uncemented and cemented THA (Fig. 2). The degree of prosthetic head acetabular coverage was also compared between the 2 groups (uncemented and cemented) both immediately and 4 months after surgery. The PHN angle was defined as the angle formed by a line connecting the centers of prosthetic and femoral heads and one drawn between the center of prosthetic head and the craniodorsal rim of reamed acetabulum (Fig. 2). All PHN angles were measured by use of the same image processing software^4^ using the “*Angle tool*”. Analyzed images and measurements were then saved for statistical analysis.

### Statistical analysis

Variables were reported as mean ± SD, and a value of *P <* .05 was considered significant. Variables of interest were compared by the use of paired, 2-tailed t-test using commercial scientific 2D graphing and statistics software^5^. A 95% confidence interval (95% CI) was calculated for selected measurements. Variables were tested for normality using D’Agostino & Pearson omnibus test before statistical analysis, and were reported to be normally distributed. The average radiographic bone GV of each and combined periprosthetic acetabular zone(s) was calculated immediately and 4 months after surgery in both groups. The calculated average bone GV of each zone, 4 months after surgery, was converted to a percentage of the immediate postoperative value. Prosthetic head Norberg (PHN) angle was calculated and compared using the same t-test.

## Results

There were no significant differences in the age and body weight between the 2 groups of dogs. The means (±SD) age and body weight were 3.5 (±1.6) years (range, 1 to 6.3 years) and 32.5 (±5.9) kg (range, 27 to 42.5 kg) in dogs with uncemented THA, respectively. The means (±SD) age and body weight were 3.0 (±2.2) years (range, 1 to 7.1 years) and 38.3 (±9.4) kg (range, 27.2 to 55.0 kg) in dogs with cemented THA, respectively. Among the dogs with uncemented THA, there were 6 females (4 spayed) and 1 intact male, and among the dogs with cemented THA, there were 2 females (1 spayed) and 5 males (3 castrated). Among a total of 22 patients underwent THA, eight dogs were excluded from the study because they had major postoperative complications (2 cases), contralateral THA (3 cases), or an evidence of pelvic tilt on the VD radiographs taken immediately or 4 months postoperatively (3 cases).

In uncemented hip arthroplasty, each of the central (pubic) and caudal (ischial) periprosthetic acetabular region (zones 2 and 3, respectively) showed a significant increase (zone 2, *P* = .021 and zone 3, *P* = .003) in the mean GV at 4 months postoperatively compared with values calculated immediately after surgery (Table 1). However, there was no significant difference (*P* = .083) in the mean bone GV of the cranial (iliac) periprosthetic acetabular region (zone 1) after 4 months of uncemented hip arthroplasty. The mean overall periprosthetic bone GV was greater (*P* = .018) after 4 months of uncemented hip arthroplasty (Table 1). There were no differences in the mean periprosthetic acetabular gray scale values for each and combined zone(s) after 4 months of cemented THA compared with those determined immediately after surgery (Table 1). The mean periprosthetic acetabular bone GV for each zone after 4 months of uncemented THA, expressed as a percentage of immediate postoperative value, markedly increased to 109% and 110% for zones 2 and 3, respectively (Table 2). However, there were no marked changes identified in these percentages for the 3 periprosthetic acetabular zones after cemented THA (Table 2).

**Table 1:** Mean (±SD) values for regional (3 periprosthetic acetabular zones) and overall mean radiographic bone GV, and prosthetic head acetabular coverage immediately and 4 months after uncemented and cemented THA (*n* = 7, each)

	Uncemented THA			Cemented THA		
Parameter	Immediately postoperative	4 months postoperative	*P*-value	Immediately postoperative	4 months postoperative	*P*-value
Zone 1 (GV)	166.3 ± 26.93	176.2 ± 26.59	0.083	166.4 ± 24.49	173.9 ± 20.74	0.243
Zone 2 (GV)	166.7 ± 31.45	181.5 ± 29.69*	0.021	171.5 ± 25.89	178.3 ± 19.29	0.292
Zone 3 (GV)	167.4 ± 26.42	183.4 ± 26.56*	0.003	164.4 ± 21.60	168.5 ± 20.04	0.449
Mean overall bone GV	166.8 ± 28.10	180.4 ± 27.45*	0.018	167.4 ± 23.82	173.6 ± 19.82	0.303
Prosthetic head GV (control)	217.3 ± 36.27	220.1 ± 39.76	0.797	220.3 ± 30.81	227.4 ± 10.09	0.279
Prosthetic head Norberg (PHN) angle (°)	112.8 ± 9.36	112.5 ± 7.67	0.851	106.9 ± 12.03	104.2 ± 14.06	0.179

*GV* gray scale value

*THA* total hip arthroplasty

*P < .05 compared with immediate postoperative value

**Table 2:** Average radiographic bone GV for each periprosthetic acetabular zone 4 months postoperatively, expressed as a percentage of immediate postoperative value

Zone	Region	At 4 months (%)^a^ (Uncemented THA)	At 4 months (%)^a^ (Cemented THA)
1	Cranial (iliac) acetabulum	106.0	104.5
2	Central (pubic) acetabulum	108.9	104.0
3	Caudal (ischial) acetabulum	109.5	102.5

*GV* gray scale value

*THA* total hip arthroplasty

^a^Percentage of immediate postoperative value

The percentages of mean bone GV for the 3 periprosthetic acetabular zones at 4 months postoperatively, expressed as a percentage of immediate postoperative value, increased significantly (*P* = .023) in dogs with uncemented THA (mean ± SD = 108.1 ± 1.90) compared with those with cemented THA (mean ± SD = 103.7 ± 1.04). The 95% CIs for the percentage of relative GV of the 3 periprosthetic acetabular zones were 103–113% for the uncemented group and 101–106% for the cemented group. In both uncemented and cemented groups, there was no difference in the area of each or combined periprosthetic acetabular zone(s) measured immediately after surgery and that measured 4 months postoperatively. The area of each prosthetic head (control) measured immediately after surgery did not differ from that measured after 4 months of surgery in both groups. Furthermore, the mean radiographic GV of each prosthetic head (control) calculated immediately after surgery did not differ from that calculated after 4 months for either implant system (Table 1).

The PHN angles measured immediately postoperatively did not change after 4 months of surgery in either uncemented (*P* = .851) or cemented (*P* = .179) groups (Table 1). Additionally, no significant differences identified in the PHN angles between the 2 groups of dogs both immediately (*P* = .328) and 4 months (*P* = .198) after surgery (Table 1). The 95% CIs for the PHN angle immediately after surgery were 104.1^o^ to 121.4^o^ for the uncemented group and 95.8^o^ to 118^o^ for the cemented group. The 95% CIs for the PHN angle 4 months postoperatively were 105.4^o^ to 119.6^o^ for the uncemented group and 91.2^o^ to 117.2^o^ for the cemented group.

## Discussion

Although Dual-energy x-ray absorptiometry (DEXA) remains the most reliable tool to evaluate periprosthetic bone remodeling after THA, via determination of bone mineral density (BMD) surrounding a prosthetic implant [12, 22, 23], lack of local DEXA availability and personal clinical expertise are still big challenges, especially in veterinary practice. Alternatively, a simply used and clinically applicable medical and radiological image processing software (ImageJ Software) has been used previously to evaluate calvarial bone density in mice [16], and recently to quantitatively assess periprosthetic femoral bone GV after THA in dogs [5]. The ImageJ processing software has thus been used in the study reported here to determine the mean radiographic bone GV of each and overall periprosthetic acetabular zone(s) after uncemented and cemented THA using digitized canine pelvic radiographs. We propose that determining the mean radiographic bone contrast (expressed as a GV) of each acetabular zone around the prosthetic implant may represent adaptive bone remodeling after THA. Assessment of the PHN angle was also achieved using the same software to evaluate the degree of prosthetic head acetabular coverage and implant stability after 4 months of uncemented and cemented THA. The PHN angle was also calculated to compare the degree of acetabular coverage between the 2 designs of hip arthroplasty both immediately and 4 months after surgery.

Four months was selected because periprosthetic bone remodeling has previously been reported to increase after 4 weeks of canine THA [14, 24]. Furthermore, in 2 recent studies, significant periprosthetic bone changes occurred during the first 3–6 months after THA in people and at 4 months postoperatively in dogs [5, 25]. In addition, the first postoperative follow-up of uncomplicated surgeries is routinely performed 4 months after surgery in our clinic. We, therefore, expected a period of 4 months after THA to be sufficient to monitor changes associated with periprosthetic acetabular bone in our subjects. Our designed 3-region of interest model was modified from a widely accepted four-region model previously used to determine periprosthetic acetabular bone remodeling after THA in people [17–19]. In the study reported here, we combined the 2 central (pubic) regions of interest (zone 1 and 2) described in the human model as a single central (pubic) region of interest (zone 2) in our canine model. Field lengths of 15 mm and 10 mm were selected to create the cranial (iliac) and caudal (ischial) regions of interest, respectively. The field lengths were configured since the cranial periprosthetic acetabular region is anatomically larger than caudal acetabular region.

Acetabular bone densification is typically evaluated radiographically as either present or absent [26–28], and planar analysis of coxofemoral joint does not provide quantitative information on bone changes [28]. We, therefore, propose that quantitative image analysis using ImageJ software may reveal subtle changes at the level of the periprosthetic acetabular bone in early stages after THA. Furthermore, periprosthetic acetabular bone changes may provide information about the efficiency of postoperative limb use via quantitative determination of the acetabular GV which reflects the amount of load-bearing applied to the prosthetic hip joint. In the present study, the increased regional bone GV around the acetabular component, most likely at the central and caudal periprosthetic acetabular regions, after 4 months of uncemented design of hip arthroplasty may be related to cancellous bone densification in these regions. This is consistent with a previous study that recorded 50 canine hips with increased bone densification around uncemented acetabular component [27]. The periprosthetic acetabular bone densification could be a manifestation of an early return to limb function after uncemented THA. We, therefore, propose that compressive weight-bearing forces may be distributed around the acetabular cup with the load bearing being more concentrated on the central and caudal regions of the acetabulum after uncemented THA in dogs. This may be explained by the tight fit of the metallic acetabular cup into the periprosthetic acetabular bone that may cause higher stress concentration created in these regions during weight bearing. The marked increase in the percentages of the mean GV for zones 2 (109%) and 3 (110%) after 4 months of uncemented THA is an evidence of relative bone densification around the corresponding prosthetic acetabular cups. These findings were also supported by the overall increase in the mean GV of the combined zones after 4 months of uncemented hip arthroplasty. The average bone GV of each and combined acetabular zone(s) did not change significantly after 4 months of cemented THA. The percentages of the mean GV for the 3 acetabular zones at 4 months postoperatively decreased significantly in dogs with cemented design of hip arthroplasty compared to those with uncemented design. This might be an evidence of the proposed higher stress concentration created along the periprosthetic acetabular bone after uncemented hip arthroplasty. However, further investigation is still necessary to prove these theories as the dogs in our study were not evaluated clinically, as well as the follow-up time was short and the impact on long-term outcome and survival was not determined after both designs of THA.

In a recent study evaluating femoral bone changes around the prosthetic femoral stem in dogs, cemented design of THA appeared more likely to improve adaptive bone remodeling around the implant 4 months after surgery [5]. In our current study, however, the adaptive bone remodeling around the prosthetic acetabular cup was relatively improved after 4 months of uncemented THA. We, therefore, propose that the survival rate of uncemented THA is expected to be superior in the acetabular components compared with that in the corresponding femoral stems. This finding is in agreement with that reported in human literature [7, 29, 30], as the survival of acetabular cups was found to be superior to that of the corresponding femoral stems after uncemented THP. In cemented design of canine THA, there were neither femoral [5] nor acetabular bone changes around the corresponding prosthetic implants after 4 months of surgery. Thus, the survival rate of cemented hip arthroplasty was relatively acceptable in both acetabular component and its corresponding femoral stem. Nevertheless, further long-term follow-up investigating the other possible factors influencing implant survival is still warranted to support our findings. The absence of osteolytic changes around our uncemented and cemented acetabular components after 4 months of surgery (indicated by lack of decreased GV after 4 months of surgery) is consistent with a previous study that found no radiographic evidence of periprosthetic acetabular bone lysis 6 months after uncemented THA in dogs, and yearly thereafter [13]. However, unlike the study reported here, long-term follow-up was achieved in this previous study and cemented THA was not investigated. Although postoperative assessment of limb function was not achieved in the present study, the 2 designs of hip arthroplasty are expected to have shown an acceptable return to limb function after 4 months of surgery. The anticipated return to limb use following uncemented and cemented THA may be explained by the radiographic findings reported in our study. This may also be supported by a recent study that reported an obvious improvement of vertical ground reaction force 4 months after uncemented and cemented THA in 24 dogs [31]. In this previous study, none of the 2 designs of THA had a greater advantage over the other with regard to the degree of lameness improvement during this short period of follow-up [31]. In general, the evidence of most of these studies indicated that proper alignment and adequate stability of the implant play a dominant role in initiating adaptive periprosthetic bone remodeling, and thereby success of THA in people and dogs [5, 7, 13, 30]. Adaptive periprosthetic bone remodeling may, however, be a temporary response and other factors could predispose to future chronic cortical bone loss secondary to surgical trauma associated with osseous reaming of canine femurs and acetabulae [14, 32]. Additionally, bone loss and aseptic loosening remain serious long-term complications associated with stable and unstable THA in dogs [2, 3]. Therefore, future long-term investigation is still warranted to evaluate the incidence of implant loosening and gait pattern after uncemented and cemented THA in a larger population of dogs. Two previous studies evaluated the load bearing areas of the acetabulae in 5 healthy cats and 4 healthy dogs [33, 34]. The caudal and central regions of the acetabulae were fully and partially load bearing, respectively, during the physiological stance phase, whereas the cranial region was non-load bearing in the 5 healthy cats [33]. However, the 3 regions of the canine acetabulae were load bearing, with the cranial and caudal regions being fully and the central being partially loaded during stance phase [34]. In people, there was an initial decrease in BMD of acetabular region 1 (resembling the cranial zone in dogs) followed by a restoration of bone density after 3 years of uncemented hip arthroplasty [17]. In human acetabular regions 2 and 3 (resembling the central zone in dogs), there was a very slight decrease in the associated BMD throughout the period of the study [17]. Whereas, in acetabular zone 4 (resembling the caudal zone in dogs), BMD increased significantly during the time of investigation [17]. We, therefore, propose that the great variation in the standing angle of the hip joint between people and dogs may result in variety in the natural load applied on the coxofemoral joint after THA. A previous study comparing hip joint forces in sheep, dog, and people also found a wide variation of load directions and magnitude among the 3 species after THA [35].

Study limitations included the small number of dogs enrolled in each group and the short follow-up period. The small sample size of subjects was related to the limited number of candidates for THA admitted to our clinic. Exclusion criteria also played a crucial role in reducing the number of enrolled dogs. Despite a control material (prosthetic head) with standard density was used on each radiograph in our study to standardize the mean GV between radiographs, the accuracy of this software, as a new method of analyzing bone changes after THA, has not been validated and is considered a limitation of our study. A future prospective study is, therefore, necessary to determine the reliability and precision of ImageJ software as a mean of calculating bone GV compared with DEXA as a mean of measuring actual BMD. Another weakness of the study is that, in cemented design of THA, the mean GV of periprosthetic acetabular bone of each zone was measured without exclusion of the associated cement which may have influenced the results of our investigation. However, in a previous human study, femoral periprosthetic BMD was precisely measured in cemented design of THA without exclusion of the cement mantle [36]. In addition, we compared the mean GV of the same region of interest with the same cement mantle for each acetabular zone immediately and 4 months after surgery, we therefore expect that any changes in the actual acetabular mean bone contrast would have been recorded by the used software. Also, the contribution of cement to the measured periprosthetic zone contrast is expected to be minimal around the acetabular component, compared with that around the femoral component. Thus, the fact that there were no significant changes recorded in the mean GV of each acetabular zone after 4 months of cemented THA may still indicate a lack of periprosthetic acetabular bone adaptation after short-term of hip arthroplasty. Nevertheless, bone-remodeling measurements performed around uncemented THA remain more precise than those made around cemented THA [19]. The dorsal border of the acetabulum, including the weight-bearing portion of the acetabular rim [37], was not evaluated in the present study, since the dorsal acetabular edge is expected to be partially reamed during surgery, and most importantly superimposed by the prosthetic implants on extended VD projection [38]. However, future assessment of periprosthetic acetabular bone GV on both VD and mediolateral projections of coxofemoral joints is strongly recommended to support the findings reported in the present study.

## Conclusions

Regional bone adaptation of canine acetabulae around prosthetic implants varies with the design of hip arthroplasty. The uncemented design of THA is expected to relatively improve acetabular bone adaptation around the implant 4 months postoperatively, compared with cemented design; however, further investigation is still necessary to substantiate this theory. There was no radiographic evidence of periprosthetic acetabular bone lucency after 4 months of uncemented and cemented THA. Both designs of THA achieved proper acetabular coverage of the associated prosthetic head 4 months postoperatively. The degree of acetabular coverage did not differ significantly between the 2 designs of hip arthroplasty. Future long-term assessment on a larger sample size is still needed to support and confirm our results.

## Abbreviations

BMD: Bone mineral density
DEXA: Dual-energy x-ray absorptiometry
FFD: focal spot film distance
GV: Grayscale value
PHN: Prosthetic head Norberg
ROI: Region of interest
THA: Total hip arthroplasty
VD: Ventrodorsal

## Footnotes

1 BFXTM, Universal Hip System, BioMedtrix, LLC, Inc., Boonton, NJ

2 CFXTM, Universal Hip System, BioMedtrix

3 dicomPACS, Version 5.2.4, Oehm und Rehbein GmbH, Rostock, Germany

4 ImageJ 1.41/Java 1.6.0_21

5 GraphPad Prism 6, California Corporation, USA
